# Sociodemographic factors and clinical presentation of women attending Cancer Detection Centre, Kolkata for breast examination

**Published:** 2020-03-19

**Authors:** Sinjini Sarkar, Dipanwita Ghosh, Sutapa Mahata, Pranab Kumar Sahoo, Asoke Roy, Manisha Vernekar, Karabi Datta, Syamsundar Mandal, Vilas D. Nasare

**Affiliations:** ^1^Department of Pathology and Cancer Screening, Chittaranjan National Cancer Institute, Kolkata, West Bengal, India; ^2^Department of Gynaecological Oncology, Chittaranjan National Cancer Institute, Kolkata, West Bengal, India; ^3^Department of Epidemiology and Biostatistics, Chittaranjan National Cancer Institute, Kolkata, West Bengal, India

**Keywords:** breast cancer, gynecologic and obstetric history, mammography, fine-needle aspiration cytology, breastfeeding history

## Abstract

**Background:**

Breast cancer is the most common cancer in Indian women.

**Aim:**

The aim of the study was to report the sociodemographic factors, habits, personal history, gynecological and obstetric history, the clinical presentation of Indian women, and analyze those factors with the diagnosis of breast cancer.

**Methods:**

This study is based on retrospective data collection from case files of women who attended the Cancer Detection Centre during January1995-September 2016.

**Results:**

Data analysis for 1196 women showed 31.5% aged between 26 and 35 years; 90.7% were Hindus; 61.3% school-educated; 77.0% housewives/unemployed; 80.6% married and 98.2% were non-vegetarian. Physical activity, medical history and gynecologic history of menarche, menstrual type, menopause, marital age, and breast feeding history had a strong correlation with clinical diagnosis (p<0.05). About 8.4% of the total population was diagnosed with breast cancer using smear cytology, FNAC, mammography, and USG.

**Conclusions:**

Age, lack of proper education, marital status, food habit, physical activity, age of menarche, menstrual type, menopause, marital age, and breastfeeding history were highlighted as significant risk factors of breast cancer in Indian women. Smears from nipple discharges, FNAC, mammography, and USG are effective methods for breast cancer detection in low-cost setting where routine organized screening programs are not available.

**Relevance for patients:**

The study will identify important risk factors among women in the Eastern region of India. Thus, background information of patients can be used to emphasize the importance of organizing breast cancer screening while making public health policies and implementing breast cancer control programs.

## 1. Introduction

According to the 2018 report of the GLOBOCAN project, breast cancer accounts for 11.6% (2.08 million) of all new cancer cases and 6.6% of all cancer deaths [1]. Its prevalence has been increasing in both developing and developed countries [2]. In India, 162 468 new cases of breast cancer and 87090 mortality were estimated in the year 2018 [3] making it the most common cancer in Indian females. The epidemiologic evidence shows that late-stage diagnosis for breast cancer is related to several of sociodemographic characteristics such as age, religion, level of education, occupation, marital status, food habit, family size, monthly income, unemployment, and family history of breast cancer [4,5]. Physical activity and smoking are modifiable risk factors that have been associated with breast cancer overall to some or larger degree [6,7]. Most of the literature reported that breast cancer is related to the reproductive life of women: early menarche, nulliparity, low parity or late age at first birth, breastfeeding history, late menopause, as well as hormonal (endogenous or exogenous) factors [8-11].

In India, the recommended screening methods for breast cancer are clinical examination, biopsy (tissue cytology and FNAC), mammography, and ultrasound. The clinical examination of breasts for abnormalities such as a lump, color change and discharge is a fundamental method for breast assessment and is used as a routine technique for breast cancer diagnosis. Cytological examination (smears) of nipple discharge/nipple retraction is performed routinely but has little complementary diagnostic value [12]. Fine-needle aspiration is rapid, less invasive, and inexpensive and plays a major role in pre-operative diagnosis of breast cancer [13]. Mammography is also one of the most effective and efficient techniques used for the detection of breast tumors with well-acceptance and improved patient adherence to the test [14]. Ultrasound is a promising adjunctive screening modality because it is widely available, relatively inexpensive, and well-tolerated by patients. Furthermore, suspicious breast lesions can be readily biopsied under ultrasound guidance [15].

The breast cancer screening guidelines have not changed for decades but there is limited data available on the Indian population from Cancer Detection Centers across the country. Therefore, this study aims to describe the association of breast cancer diagnosis with demographic characteristics, personal history, gynecological and obstetric history, and clinical presentation of women.

## 2. Materials and Methods

### 2.1. Study population and data collection

The study population includes women who attended the Cancer Detection Centre at Chittaranjan National Cancer Institute, Regional Cancer Centre, Kolkata, India. All women were 18 years or older who were examined for malignancy of the breast. The data set comprises of demographic information such as age, religion, education, occupational status, marital status, food habit, family size, monthly income, and physical activity; habits such as betel leaf, nut lime, dokta, jarda, catechu, guraku, gutka, snuff, cigarettes, bidi, and chewing tobacco; gynecologic and obstetric history; symptoms/complaints; clinical examination of breasts; and suspected clinical diagnosis and follow-up. The gynecological characteristics of the breast cancer screening participants were recorded with information such as the age of menarche, the regularity of menstrual cycle, and menopause. Similarly, the obstetric history recorded data on marital age, parity, abortion, type of child delivery, and breastfeeding history. Techniques such as smears from nipple discharges, FNAC, mammography (for patients >40 years), and USG were applied for detection of cancer. All the information was recorded in hand by the attending physicians and later the data were abstracted, analyzed, and re-entered in the database.

### 2.2. Study design

This study design is based on retrospective data collection (January 1995-September 2016) from the case files of female participants. Our study has included case records of patients with symptoms such as breast pain/mass, nipple discharge, nipple/skin retraction, axillary mass/pain, or others; who underwent a clinical examination of either or both the breasts, surrounding area, and discharges. Participants with age below 18 years and those with different symptoms and different cancer diagnoses were excluded from this study.

### 2.3. Statistical analysis

Statistical analysis was performed with the help of Epi Info (TM) 7.2.2.2 which is a trademark of the Centers for Disease Control and Prevention (CDC) and SPSS16. Using this software, basic cross-tabulation and frequency distributions were prepared. Corrected Pearson Chi-square (χ^2^) was used to test the association between different study variables in case of one of the cell frequencies found to be <5-12. The significance level was set at 0.05 with 95% confidence interval. *P* ≤ 0.05 was considered to be statistically significant.

## 3. Results

### 3.1. Description of the cohort

A total of 1196 case records were found to be eligible for the study. The suspected clinical diagnosis reports 1034 (87.5%) cases to be benign, 42 (3.5%) to be inflammation of breast, and 100 (8.4%) malignant. The overall mean age of the participants was 37.32 ± 12.79 (mean ± SD) years with most of them belonging to the age group of 26-35 years. About 90.7% were Hindus and 9.3 % were Muslims. Among all, 26.4% were graduates, 61.3% had a primary/secondary education, and illiterate constitutes 13.6%. Further analysis revealed a higher participation rate of housewives/unemployed (77%); while 14.4% were unmarried and 4.8% were widowed/divorced/separated. Participants were mostly (98.2%) non-vegetarians with only 1.8% vegetarians. Addictions of betel leaf/nut lime/dokta/guraku/gutkha/smoking/chewing tobacco were noted. 70 had a history of betel leaf/nut lime/dokta addictions, 68 addicted with guraku/gutkha, and 19 women had a habit of smoking/chewing tobacco. Personal history of physical activity (sedentary 20.1%; moderate79.9%); family history of cancer (breast cancer 2.3% and other types of cancer 19.1%); past medical history (X-ray chest 13.6%; breast surgery 0.4% and ligation11.7%), and contraceptive usages (16.1%) was recorded. The family size and monthly income are mentioned in the demographic characteristics of the participants (Table 1). Data on age of menarche and menstrual type were found for 1167 women indicating that 1.6% had menarche below the age of 10 years, 45.9% had menarche between 10 and 13 years, 52.4% having menarche above 13 years and, among them, only 9.4% mentioned irregular periods. Participants consisted of 228 (19.3%) were post-menopausal and 952 (80.6%) were pre-menopausal women. The highest number of the population was married between the ages of 18 and 23 years followed by 303 participants, who were married below the age of 18 years. 304 participants were nulliparous, 477 had 1-2 children, 339 had 3-5 children, and 60 had more than 6 children. 738 (83.1%) women had vaginal child birth while 150 underwent surgery. 237 participants had a history of abortions. Case records showed 7.7%, 17.8%, 27.9%, and 19.7% of women having breastfeeding history for <6 months, 6-12 months, 2-5 years, and more than 5 years, respectively. Three hundred and twelve women had no breastfeeding history. The participants presented with symptoms such as breast mass (44.5%); breast pain (34.3); nipple discharge (5.8%); nipple or skin retraction (4.1%) axillary mass or pain (2.6%); and others (7.8%). Smears for nipple and discharges were performed for 120 (10.1%) cases, FNAC performed for 876 (74.2%), USG of 991 (83.9%) breasts and mammography of 61 (5.1%) breasts was done. Among the participants, doctors referred 107 patients to hospital; prescribed to 37, and recommended routine checkup for 134.

**Table 1:** Descriptive statistics of characteristics assessed in the study subjects.

Characteristics of participants (*n*=1196)	Subgroups	Frequency (%)
Age (years)	18-25	245 (20.4)
	26-35	377 (31.5)
	36-45	309 (25.8)
	46 above	265 (22.1)
Religion (*n*=1180)	Hindu	1071 (90.7)
	Muslim	109 (9.3)
Education	Illiterate	161 (13.6)
	School education	724 (61.3)
	Graduates and above	311 (26.4)
Occupation	House wife/unemployed	909 (77.0)
	Service/retired	102 (8.6)
	Students	101 (8.5)
	Farmers/labors	84 (7.1)
Marital status (*n*=1180)	Unmarried	171 (14.4)
	Married	952 (80.6)
	Widowed/divorced/separated	57 (4.8)
Food habit	Vegetarian	20 (1.8)
	Non-vegetarian	1160 (98.2)
Family size	1-4	715 (60.5)
	5-10	449 (38.0)
	10+	32 (2.7)
Monthly income per month (Rupees)	<Rs 1000/-	118 (9.9)
	>Rs 1000 to Rs 5000/-	673 (57.0)
	>Rs 5000 to Rs 10000/-	188 (15.9)
	Rs 10001/- and above	217 (18.3)
Menarche (*n*=1167)	<10 years	19 (1.6)
	10-13 years	536 (45.9)
	13+ years	612 (52.4)
Menstrual type (*n*=1167)	Regular	1058 (90.6)
	Irregular	109 (9.4)
Menopausal women (*n*=1180)	No	952 (80.6)
	Yes	228 (19.3)
Marital age (*n*=1180)	Unmarried	175 (14.8)
	<18 years	303 (25.6)
	18-23 years	490 (41.5)
	24 years and above	212 (17.9)
Parity (*n*=1180)	Nil	304 (25.7)
	1-2	477 (40.4)
	3-5	339 (28.7)
	6 and above	60 (5.0)
Abortion (*n*=1180)	No	943 (79.9)
	Yes	237 (20.0)
Delivery (*n*=888)	Normal	738 (62.5)
	Operative	150 (12.7)
Breast feeding history (*n*=1178)	<6 months	92 (7.7)
	6-12 months	211 (17.8)
	2-5 years	330 (27.9)
	>5 years	233 (19.7)
	Nil	312 (26.4)
Chief complaints	Breast mass	533 (44.5)
	Breast pain	411 (34.3)
	Nipple discharge	70 (5.8)
	Nipple or skin retraction	50 (4.1)
	Axillary mass or pain	38 (2.6)
	Others	94 (7.8)
Clinical examination of breast	Nil	30 (2.5)
	Right breast	388 (32.8)
	Left breast	339 (28.7)
	Both	409 (34.6)
	Nipple discharge	8 (0.6)
Clinical diagnosis	Benign	1034 (87.5)
	Inflammation	42 (3.5)
	Malignant	100 (8.4)
Smear	Yes	120 (10.1)
Smear results	Normal	12 (0.1)
	Mild	56 (46.6)
	Moderate	38 (31.6)
	Carcinoma	16 (13.3)
FNAC	Yes	876 (74.2)
FNAC results	Normal	229 (26.1)
	Benign	576 (65.7)
	Carcinoma	68 (7.7)
Mammography	Yes	61 (5.1)
USG of breast	Yes	991 (83.9)
Type of follow-up	Nil	13 (1.1)
	Doctor prescribed	37 (3.1)
	Routine checkup	134 (11.3)
	Discontinued	894 (75.7)
	Referred to hospital	107 (9.0)
Follow-up	Within 3 months	106 (8.9)
	3-12 months	49 (4.1)
	1-3 years	17 (1.4)

### 3.2. Association between sociodemographic characteristics and detection of cancer

The associations between age (*P*=0.001), lower education (*P*=0.001), marital status (*P*=0.001), and food habit (*P*=0.008) with the clinical diagnosis of cancer were highly significant (Table 2). No significance was found with the habits and clinical diagnosis (*P*=0.916). Physical activity (*P*=0.001) and past medical history (*P*=0.011) were significant with breast cancer diagnosis. Our study failed to find a risk of family history of cancer (*P*=0.835) among the patients and no genetic testing was carried out. Contraceptive usage (*P*=0.100) was also not significantly associated with clinical diagnosis (data not shown).

**Table 2:** Association of demographic characteristics with clinical diagnosis.

Clinical diagnosis	Outcome	Demographic characteristics															

		Age (years)				Education			Occupation				Marital status			Food habit	
				
		18-25	26-35	36-45	46 and above	Illiterate	Primary schooling	Secondary schooling	Unemployed/housewife	Service/retired	Student	Others	Unmarried	Married	Widowed/divorced	Veg	Non-veg
	Benign	232	347	259	205	127	630	286	775	94	98	76	160	827	40	13	1014
	Inflammation	11	15	19	8	9	32	12	43	4	2	4	7	44	2	2	51
	Malignant	2	15	31	52	25	62	13	91	4	1	4	4	81	15	5	95
Total		245	377	309	265	161	724	311	909	102	101	84	171	952	57	20	1160
*P*-value		*P*=0.001				*P*=0.001			*P*=0.015				*P*=0.000			*P*=0.008	

### 3.3. Association between gynecological and obstetric history and detection of cancer

**Figure 1:**
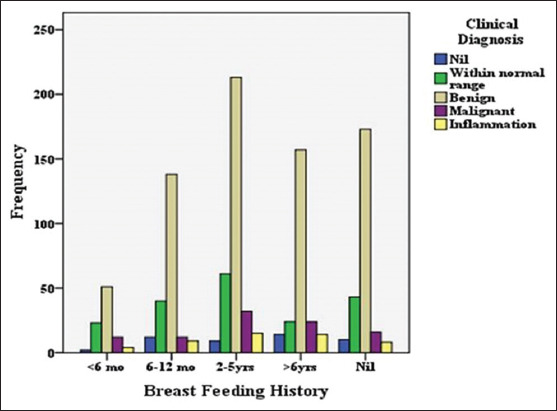
Frequency of clinical diagnosis with breast feeding history (χ^2^ = 27.098; *P* = 0.040).

**Figure 2:**
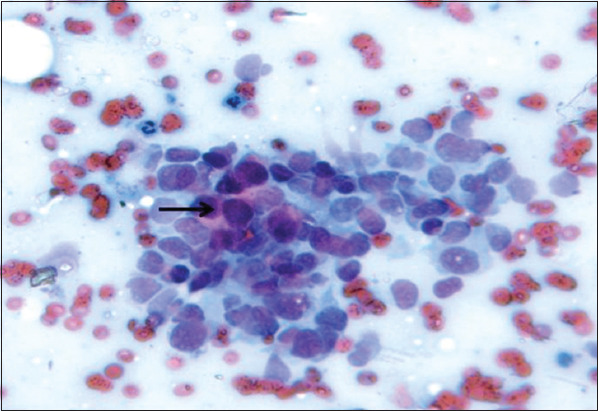
Discohesive sheets and clusters of malignant epithelial cells showing carcinoma breast (black arrow).

Data analysis shows more incidences of cancer in patients with early age menarche (<13 years). Among the participant population, 21.3% of the post-menopausal women were diagnosed with cancer whereas only 5.3% of pre-menopausal women had cancer. Further analysis shows that 13% of women with breast feeding history of less 6 months were diagnosed with breast cancer while that percentage is 9.9 in the group with 2-5 years or more of breastfeeding history. The least incidence (5.6%) of breast cancer was observed in women of the group with 6-12 months of breastfeeding (Figure 1). Menarche (χ^2^=25.804; *P*=0.001), menstrual type (*P*=0.048), menopause (χ^2^=68.155; *P*=0.0001), and marital age (*P*=0.025) and breastfeeding history (*P*=0.040) were highly significant with clinical diagnosis (Table 3). There were no associations with parity, (*P*=0.111), abortion (*P*=0.895), and type of delivery (*P*=0.394) with clinical diagnosis. From the clinical diagnosis, 48 cases of malignancy were presented with breast mass and the least number (4) of cancer cases was presented with axillary mass or pain. From smears, 16 cancer cases were identified; FNAC identified 68 cases (Figure 2); and mammography diagnosed 14 cases and 94 cases of cancer were identified by ultrasound.

**Table 3:** Association of gynecologic history with clinical diagnosis,

Clinical diagnosis	Outcome	Gynecologic history										

		Menarche			Menstrual type		Menopause		Marital age			
			
		<10 years	10-13 years	>13 years	Regular	Irregular	Yes	No	Unmarried	<18 years	18-23 years	24 years and above
	Benign	17	468	531	918	98	856	172	166	258	422	182
	Inflammation	1	16	34	47	5	45	8	6	15	25	6
	Malignant	1	52	47	93	6	51	49	3	30	43	24
Total		19	536	612	1058	109	952	229	175	303	490	212
*P*-value		*P*=0.001			*P*=0.048		*P*=0.000		*P*=0.025			

Records of follow-up within 3 months, 3-12 months and more than 1-3 years were found for only 106, 49, and 17 patients, respectively. The maximum number of the total participant population (894, 74.7%) discontinued any kind of follow-up (Table 1).

## 4. Discussion

The relationship between the socioeconomic status of patients and breast cancer is complex [16]. In the present analysis, we observed that the maximum of participant population comprised women of ages between 26 and 35 (31.5%) years; 90.7% Hindus; 61.3% school educated, 77% housewives/unemployed; 79.7% married; and mostly non-vegetarians (98.2%). The associations between age, education, occupation, marital status, and food habit with the clinical diagnosis were highly significant (*P*=0.001). In India, Sathwara *et al*. 2017 reported that sociodemographic factors such as age, religion, marital status, and occupation were not found to be significantly associated with stage at presentation but the level of education was highly associated with diagnosis of breast cancer at hospital-based cancer registry, Tata Memorial Hospital (TMH), Mumbai, India. Previous studies have demonstrated that lower education and income are important causes of delay in the diagnosis of breast cancer in women in developing countries [17-19].

In our study, physical activity and past medical history were significantly associated with the clinical diagnosis. However, the present study has failed to show associations between family history of cancer and contraceptive usage with the clinical diagnosis of breast cancer. Another study reported from Turkey revealed that the family history for breast cancer risk increases 5.7 times in a woman who has a first-degree relative suffering from breast cancer [20]. Oral contraceptive uses were also strong risk factors in Turkish women [21]. In our scenario, the contradiction may be attributed to the lower economic strata and low education level of maximum participants who might be unable to afford contraceptive medications and be unaware of their family history of cancer.

Consistent with other studies, we found that early age of menarche [22], late age of menopause [23], and breastfeeding history [24] were highly significant with breast cancer development whereas no associations were found with medical/spontaneous abortion and type of delivery [25]. Again Khalis *et al.*, 2018 found no risk associated with the menstrual type and breast cancer contradicting our results which show a significant association of menstrual type and marital age. There were no associations with parity, unlike the studies that reported decrease in breast cancer risk with increased number of live births [26,27]. This study lacks information regarding the stages of cancer at diagnosis.

Early breast cancer detection improves survival and reduces medical costs [28,29]. The story of breast cancer screening in India is also a non-existing reality. There are much social taboos associated that keeps the women from coming to diagnostic centers. The topic of breast cancer is not discussed openly in the unaware society, the stigma of being rejected by partner and potential fear of loss of the organ is among many obstacles to early diagnosis of breast cancer [30]. The most appropriate screening method for Indian women is clinical breast examination by female physicians or trained health workers. In a limited resource setting, breast ultrasound is a useful diagnostic work-up along with clinical examination. Fine-needle aspiration or core needle biopsies along with proper follow-up are the prerequisites for prompt detection and treatment. Association between patient age and screening mammography performance metrics in women 40 years or older has been evaluated using large-scale evidence from the National Mammography Database (NMD) [31]. The outcome of this study indicated no specific age cutoff point for screening and supports guidelines for encouraging screening based on of individual patient values, comorbidities, and health status. The yield of cancer diagnosed in women under the age of 40 years is considerably low than with women of ages more than 40 years. This is because younger women have denser breasts and increased breast tissue density decreases the test sensitivity. Affordability of mammography and risk of increased false positives are the major concerns with mass mammographic screenings in a country like India, where the majority of breast cancer patients are younger women. Thus, cancer detection will be lower with mammographic screening in India when compared to other countries [28,32,33].

## 5. Conclusion

Our study findings indicate age, lack of proper education, marital status, food habit, physical activity, age of menarche, menstrual type, menopause, marital age, and breastfeeding history to be associated with increased risk of breast cancer in Indian women. Significant benefits of screening have been observed in developed countries. Factors such as age shift (younger women of 30s and 40s being diagnosed), aggressiveness in younger women, increasing incidence, late presentation, and unawareness make breast cancer screening extremely important in India. Cytology smears, mammography, USG, and FNAC are effective screening methods and this strategy can prove to be useful in down-staging the disease leading to curative treatment.
